# Nutritional Risk in Oral Surgery Inpatients: Insights from a Retrospective Analysis Using Nutritional Risk Screening-2002

**DOI:** 10.31662/jmaj.2025-0343

**Published:** 2026-01-14

**Authors:** Hiroshi Kusunoki, Noriko Yasuda, Kaname Tsuji, Sho Mitsugi, Takayuki Kusunoki, Haruka Shigemura, Teruko Kawahara, Miyu Matsui, Ayumi Saito, Masanari Shimoda, Hideo Shimizu

**Affiliations:** 1 Nutrition Support Team, Osaka Dental University Hospital, Osaka, Japan; 2 Department of Internal Medicine, Osaka Dental University, Hirakata, Japan; 3 First Department of Oral and Maxillofacial Surgery, Osaka Dental University, Hirakata, Japan; 4 Second Department of Oral and Maxillofacial Surgery, Osaka Dental University, Hirakata, Japan; 5 Department of Geriatric Dentistry, Osaka Dental University, Hirakata, Japan

**Keywords:** nutritional risk screening (NRS-2002), oral and maxillofacial surgery, malnutrition, nutritional assessment, perioperative care

## Abstract

**Introduction:**

Malnutrition is common and often goes unrecognized among hospitalized patients, particularly older adults. Early identification and individualized nutritional interventions are essential for improving outcomes. The Nutritional Risk Screening 2002 (NRS-2002) is a validated tool widely used in acute care settings, but its utility in dental and oral surgery remains underexplored. This study aimed to evaluate the clinical utility of NRS-2002 in patients admitted to the Department of Oral and Maxillofacial Surgery (OMFS) at a university-affiliated dental hospital in Japan, and to examine the relationship between nutritional risk and clinical factors such as age, body mass index (BMI), diagnosis, and length of hospital stay.

**Methods:**

A retrospective observational study was conducted on 548 patients (224 men, 324 women) hospitalized between August 2024 and March 2025. Nutritional screening was performed at admission using NRS-2002. Patients scoring ≥3 were classified as at nutritional risk and further assessed according to the Global Leadership Initiative on Malnutrition criteria.

**Results:**

The overall prevalence of nutritional risk, defined as NRS-2002 ≥3, was 1.6% (n = 9), which was insufficient for robust statistical analysis. Most patients identified as at nutritional risk were elderly women with low BMI and severe conditions, including medication-related osteonecrosis of the jaw and osteomyelitis. These patients experienced prolonged hospital stays and were more likely to require oral nutritional supplements. In contrast, the majority of inpatients were younger adults, accounting for the overall low prevalence of nutritional risk.

**Conclusions:**

The NRS-2002 effectively identified older, underweight patients who underwent OMFS with severe conditions and longer hospital stays, despite the overall low prevalence of nutritional risk. Early nutritional screening with standardized tools such as NRS-2002 may help optimize perioperative management.

## Introduction

Preoperative nutritional status is a critical determinant of postoperative outcomes, including complications, length of hospital stay, and mortality, and its impact is particularly pronounced in older hospitalized patients. According to nationwide Japanese data, approximately 42% of hospitalized older adults are at nutritional risk, and 26% are classified as malnourished ^(1), (2)^.

However, these findings are based on general inpatient populations, and data specific to patients admitted for oral and maxillofacial surgery (OMFS) remain limited. Malnutrition impairs wound healing, immune function, and the response to surgical stress, thereby increasing the risk of infections and secondary organ dysfunction ^(3)^. Nutritional management is considered one of the key factors for improved and faster recovery following orthognathic surgery for jaw deformity ^(4)^. Protein status, often assessed by serum albumin, is a key indicator of tissue repair and recovery. OMFS procedures are highly invasive and anatomically constrained, frequently necessitating enteral or parenteral nutritional support. Furthermore, postoperative decline in masticatory and swallowing function exacerbate the risk of malnutrition, underscoring the need for individualized nutritional interventions ^(5)^.

The high prevalence and clinical impact of malnutrition among surgical patients have been documented in multiple studies. In Norway, an 11-year study involving 18,933 patients reported that 27% were at nutritional risk, with higher rates observed among older adults ^(6)^. In Japan, a prospective study of older patients with head and neck cancer demonstrated that a low Geriatric Nutritional Risk Index (<100) was independently associated with reduced overall survival, irrespective of age or treatment intensity ^(7)^. These findings collectively emphasize the importance of nutritional risk assessment and management in surgical care.

Evidence from Japan has also demonstrated the effectiveness of nutritional management in patients undergoing oral surgery or experiencing swallowing disorders. Compared with the conventional diet, the newly developed diet significantly reduced the time from initiation of oral intake to discharge and showed a trend toward fewer cases of aspiration pneumonia. These findings indicate that the new diet is a safe and effective approach to facilitating swallowing rehabilitation ^(8)^.

In addition, studies in community-dwelling older adults have demonstrated that reduced tongue pressure and swallowing dysfunction are associated with malnutrition, with inadequate protein intake and reduced dietary diversity identified as major risk factors ^(9)^. The oral function tests most strongly associated with malnutrition are occlusal force and tongue pressure, both of which have been reported as potential tools for assessing patients’ nutritional status ^(10)^.

The Nutritional Risk Screening 2002 (NRS-2002) is a validated tool for assessing nutritional risk in acutely hospitalized adults, and its utility has been reported across diverse surgical settings ^(11)^. The NRS-2002 is a widely used and reliable screening tool, primarily applied to adult patients in acute care hospitals. Its clinical utility has been demonstrated in various surgical settings ^(12), (13), (14), (15), (16)^. Recent studies have reported that supervised machine learning algorithms are effective in predicting postoperative malnutrition in oral cancer patients, as evaluated using the NRS-2002 ^(17)^.

At Osaka Dental University Hospital, all OMFS inpatients undergo nutritional screening using NRS-2002, with additional evaluation by the Global Leadership Initiative on Malnutrition (GLIM) criteria for those identified as at risk. This retrospective observational study was conducted under the guidance of a multidisciplinary Nutrition Support Team (NST). The objectives were to clarify the prevalence and clinical characteristics (e.g., age, body mass index [BMI], diagnosis) of nutritional risk among OMFS inpatients; to examine the association between admission malnutrition risk as determined by NRS-2002 scores and clinical outcomes (length of stay, use of oral nutritional supplements [ONS]); and to analyze the characteristics of patients with NRS-2002 ≥3. The ultimate goal was to inform early screening and the development of individualized nutritional intervention strategies. Appropriate assessment and management of nutritional risk in OMFS inpatients have the potential to reduce postoperative complications, shorten hospital stays, and ultimately improve patient outcomes.

## Materials and Methods

A retrospective observational study was conducted on 548 patients (224 men and 324 women) who were admitted to the Department of OMFS at Osaka Dental University Hospital between August 2024 and March 2025. Nutritional risk was assessed upon admission using the NRS-2002. The NRS-2002 is a validated screening tool developed by the Working Group of the European Society for Clinical Nutrition and Metabolism (ESPEN) through a systematic review of randomized controlled trials worldwide. It is based on clinical evidence and offers the advantages of simplicity and non-invasiveness. The tool evaluates nutritional risk through three components: impaired nutritional status (assessed via unintentional weight loss, reduced food intake, and BMI), disease severity, and age (an additional point is added for patients aged ≥70 years). Each component is scored from 0 to 3, producing a total score from 0 to 7. A score of ≥3 indicates that the patient is at nutritional risk ^(11)^. An overview of patient characteristics was analyzed based on this classification.

To visualize the distribution of key variables such as age, BMI, and length of hospital stay, histograms were created. Furthermore, stacked bar charts were used to examine the distribution and trends of patient subgroups, categorized by disease type and sex. Age, BMI, length of hospital stay, and sex distribution were compared between patients identified as being at nutritional risk by the NRS-2002 and those without nutritional risk.

The study protocol was approved by the Ethics Committees of Osaka Dental University. All procedures involving human participants were performed in accordance with the ethical standards of the institutional and/or national research committee where the studies were conducted (Institutional Review Board approval number 111404 at Osaka Dental University) and with the 1964 Declaration of Helsinki and its later amendments or comparable ethical standards. The requirement for written informed consent was waived, as participants were given the opportunity to opt out due to the retrospective nature of the study.

### Statistical analysis

The results are expressed as the means ± standard deviations. For intergroup comparisons, Student’s *t*-test was used for data analysis. Categorical variables are presented as absolute numbers (n) and relative frequencies (%), and were analyzed using the Fisher’s exact test. Data analysis was conducted using JMP version 17.1 software, with statistical significance set at p < 0.05.

## Results

An overview of patient characteristics is presented in Table 1. The mean age of the total cohort was 35.1 years, with women (34.4 years) tending to be slightly younger than men (36.3 years). There were notable sex differences in anthropometric measurements: the average height, weight, and BMI in men were all higher than those in women. The mean length of hospital stay was 6.2 days, with women showing a slightly longer average duration than men (6.5 vs. 5.6 days).

**Table 1.** Oral and Maxillofacial Surgery Inpatients (August 2024-February 2025).

			Total (n = 548)	Men (n = 224)	Women (n = 324)	p-Value
Age (years)*			35.1 ± 17.0	36.3 ± 17.2	34.4 ± 16.8	0.200
Height (cm)*			162.8 ± 10.7	169.5 ± 10.8	158.3 ± 7.8	**<0.001**
Weight (kg)*			59.9 ± 14.4	68.1 ± 14.9	54.2 ± 10.9	**<0.001**
Body mass index (kg/m^2^)*			22.4 ± 4.2	23.6 ± 4.4	21.6 ± 3.8	**<0.001**
Length of hospital stay (days)*			6.2 ± 4.8	5.6 ± 4.0	6.5 ± 5.3	**0.026**
Purpose of hospitalization	Jaw deformity	Orthognathic surgery, n (%)†	157 (28.6)	48 (21.4)	109 (33.6)	**0.002**
		Plate removal, n (%)†	80 (14.6)	23 (10.3)	57 (17.6)	**0.019**
	Tooth extraction, n (%)†		118 (21.5)	47 (21.0)	71 (21.9)	0.833
	Cystic lesion, n (%)†		115 (21.0)	71 (31.7)	44 (13.6)	**<0.001**
	Tumor	Malignant tumor, n (%)†	9 (1.6)	5 (2.2)	4 (1.2)	0.497
		Benign tumor, n (%)†	21 (3.8)	10 (4.5)	11 (3.4)	0.652
	Others	Inflammatory disease, n (%)†	22 (4.0)	8 (3.6)	14 (4.3)	0.826
		Mucosal disease, n (%)†	8 (1.5)	5 (2.2)	3 (0.9)	0.282
		E.g., trauma, foreign body, etc., n (%)†	18 (3.3)	7 (3.1)	11 (3.4)	1.000
NRS-2002 Score ≥3, n(%)†			9 (1.6)	2 (0.9)	7 (2.2)	0.321

P-values for comparisons of study population characteristics between men and women were calculated using Student’s *t*-test. Categorical variables are presented as absolute numbers (n) and relative frequencies (%), and were analyzed using the Fisher’s exact test. NRS-2002: Nutritional Risk Screening 2002. The boldface indicates statistically significant values. *Presented as the mean ± standard deviation. †Presented as n (%).

The most common reason for hospitalization was orthognathic surgery for jaw deformity (28.6%), representing nearly one-third of all cases. In these patients, plate removal surgery is typically performed 6 months to 1 year after the initial orthognathic procedure. This procedure, classified as jaw deformity (plate removal), represented 14.6% of all admissions. Other common reasons for admission included tooth extraction (21.5%) and cysts (21.0%), with these three diagnoses together comprising approximately 80% of all cases. A sex-based analysis revealed that orthognathic surgery for jaw deformity was more frequent in women (33.6%), whereas cysts were more prevalent in men (31.7%). Less common reasons for admission included inflammatory diseases (4.0%), benign tumors (3.8%), malignant tumors (1.6%), mucosal diseases (1.5%), and other causes such as trauma or foreign bodies (3.3%). A total of nine patients (1.6% of the cohort) were identified as being at nutritional risk, including seven women and two men.

**Figure 1.**
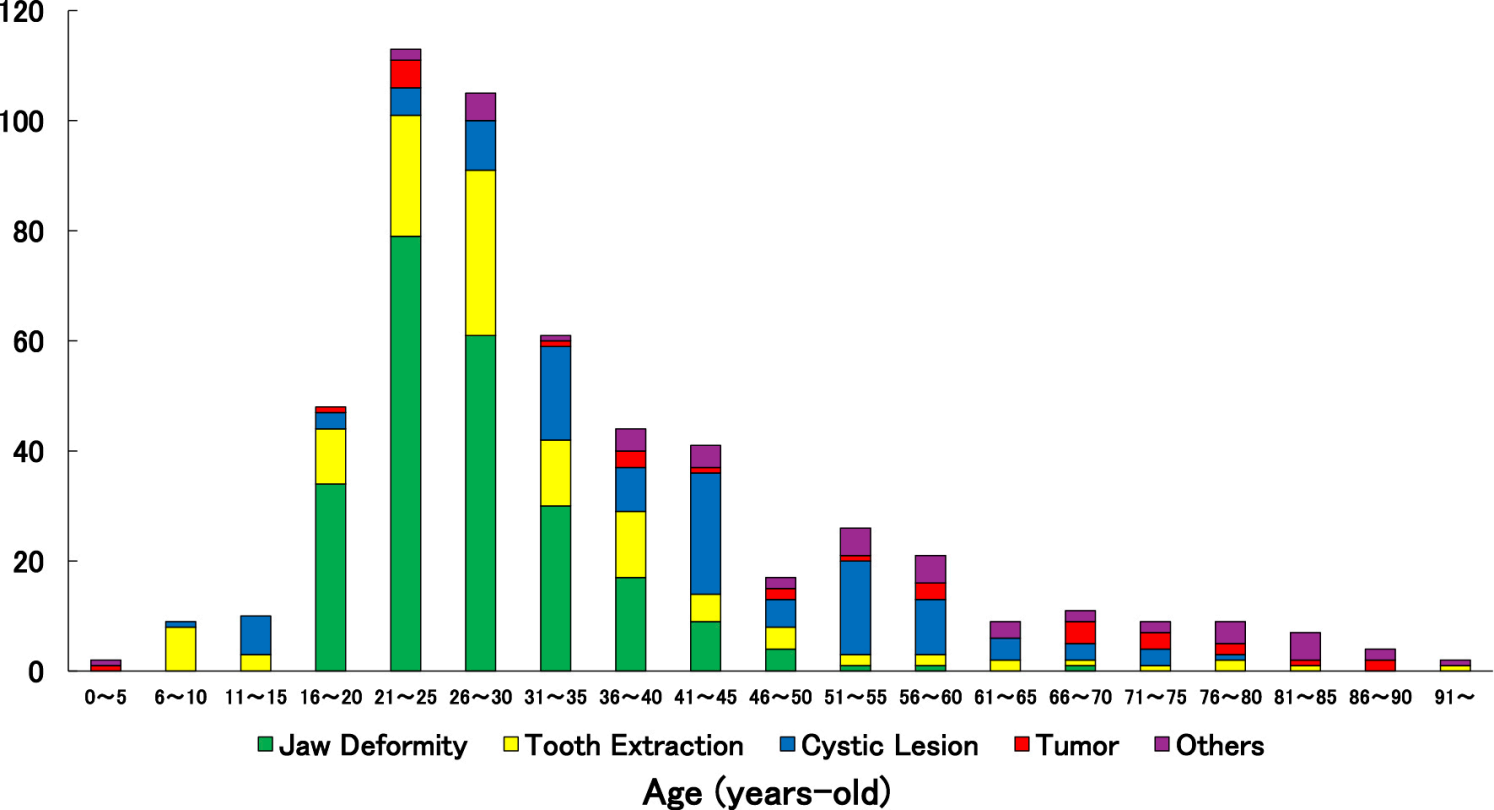
Relationship between age distribution and disease categories.

Figure 1 illustrates the distribution of disease categories across different age groups. Jaw deformities and related procedures (e.g., plate removal) were predominantly observed in younger patients, particularly those in their late teens to 20s. In contrast, conditions such as cysts, inflammatory diseases, malignant tumors, and medication-related osteonecrosis of the jaw (MRONJ) were more frequently found in older adults, indicating a clear age-related trend in disease prevalence.

**Figure 2.**
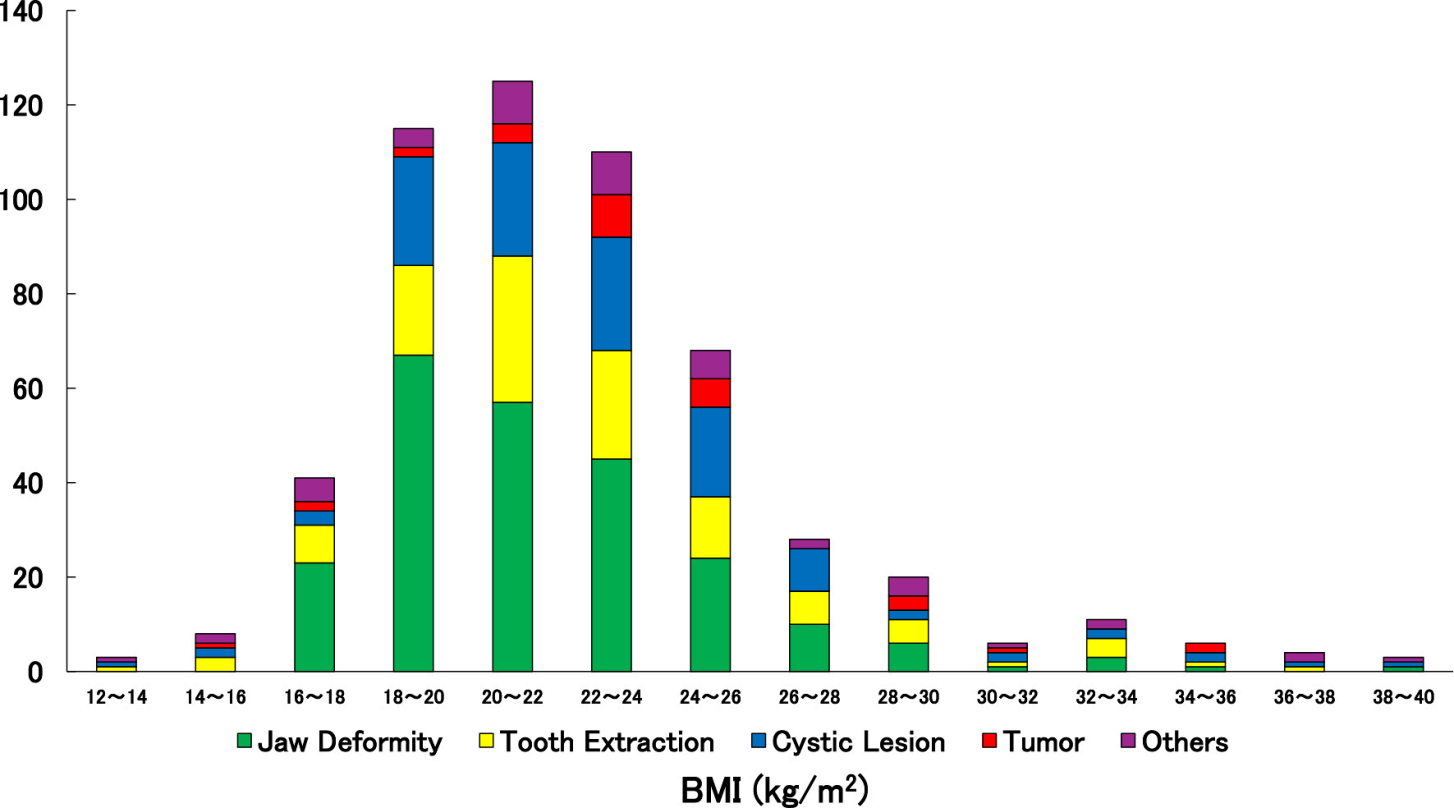
Relationship between BMI distribution and disease categories. BMI: body mass index.

Figure 2 shows the relationship between BMI distribution and reason for hospitalization. While most patients had BMIs within the normal range (18.5-25.0 kg/m^2^), a number of patients exhibited either significantly low or high BMIs. In particular, underweight status (BMI <18.5 kg/m^2^) was observed among older patients with severe conditions such as malignant tumors or osteomyelitis, suggesting a potential association with nutritional risk.

**Figure 3.**
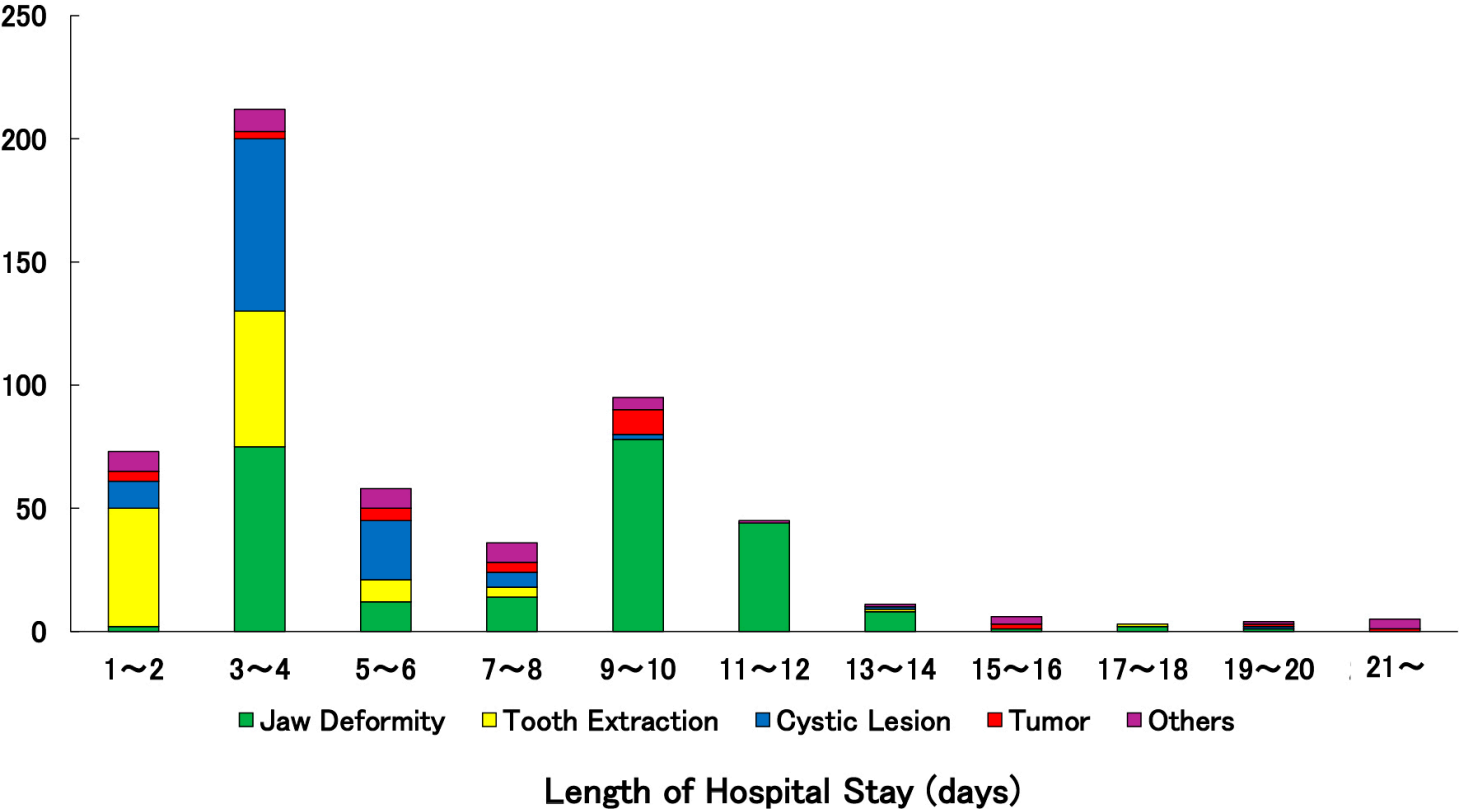
Relationship between distribution of length of hospital stay and disease categories.

Figure 3 presents the distribution of hospital stay duration in relation to disease categories. Length of stay ranged from 1-2 days to over 21 days. The most frequent duration was 3-4 days, followed by 9-10 and 1-2 days, indicating that short to mid-term hospitalization was common. However, prolonged stays (≥10 days, and occasionally over 20 days) were seen in more severe cases, such as those involving osteonecrosis or osteomyelitis. Short stays were typical for cases involving tooth extraction and mucosal lesions.

Table 2 summarizes the clinical characteristics of patients with an NRS-2002 score of ≥3, indicative of nutritional risk. As described previously, a total of nine patients were identified as being at nutritional risk, including seven women and two men. These patients tended to be older female adults with low BMI and serious conditions. Specific examples include a 91-year-old woman with MRONJ who had a hospital stay of 63 days, and an 85-year-old female with osteomyelitis of the mandible and a BMI of 12.8 kg/m^2^. Most of these patients had BMIs below 18.5 kg/m^2^ or were markedly underweight, and their hospital stays tended to be prolonged. Among them, only three of the nine patients received ONS during hospitalization. Furthermore, only three patients were formally diagnosed with malnutrition based on the GLIM criteria, despite having an NRS-2002 score indicating nutritional risk.

**Table 2.** NRS-2002 Score ≥3 at the Time of Admission Screening.

Case	NRS-2002 Score	Diagnosis	Height (cm)	Weight (kg)	Body mass index (kg/m^2^)	Length of hospital stay (days)	Malnutrition according to GLIM criteria	Treatment details
73M	3	Persistent oral bleeding after scaling	171.8	50.8	17.2	7	Applicable	The diet was gradually modified to easier-to-swallow consistencies according to the patient’s condition. To ensure adequate nutritional intake, oral nutritional supplements (ONS) were added to each meal.
83F	3	Cellulitis at left mandible	144.5	46	22.0	4	Not applicable	The patient was admitted on an emergency basis due to inflammation. With improvement in symptoms through treatment, the patient was able to consume over 50% of a soft diet consisting of porridge and finely chopped foods at discharge.
37F	3	Fibrous dysplasia of right mandible	165	60	22.0	15	Not applicable	To stimulate appetite, seaweed paste was added to the staple food. Additionally, one Calorie Mate jelly (200 kcal) was provided with breakfast.
59F	3	Cellulitis at oral floor	149.5	49.8	22.3	7	Not applicable	Due to inflammation, emergency admission was required. Oral intake remained poor; thus, one serving of a concentrated ONS (200 kcal) was added to each meal of porridge and finely minced foods. As intake did not improve, the meal texture was further softened to rice gruel and a mousse-type side dish, with Calorie Mate jelly (200 kcal) added to each meal. With a decline in inflammatory markers, food intake improved, accompanied by weight gain.
85F	3	Osteomyelitis of mandible	155	30.7	12.8	25	Applicable	Anti-inflammatory treatment was initiated. ONS (200 kcal) was added to each meal of porridge and chopped foods. The patient’s oral intake was favorable, with a 2.6 kg weight gain compared to admission.
83F	3	Cellulitis at oral floor	148	40.9	18.7	5	Applicable	The patient was admitted emergently due to inflammation and was able to consume 80-100% of a regular diet.
74M	4	Sinusitis at left side	160	48.8	19.1	3	Not applicable	The patient had diabetes and was treated with insulin. A diabetic diet of 1,400 kcal was fully consumed, and blood glucose levels remained stable throughout hospitalization.
91F	4	Medication-Related Osteonecrosis of the Jaw (MRONJ)	143	44.8	21.9	63	Not applicable	During a prolonged hospitalization of approximately two months, serum total protein levels declined postoperatively. However, one ONS was added to each meal, and oral intake was maintained. Meal texture was gradually upgraded based on the patient’s condition. At discharge, the patient was able to consume soft rice and soft vegetables, with body weight maintained.
75F	4	Osteomyelitis of mandible	152	42.5	18.4	10	Applicable	Due to hyponatremia, pickled plum paste was added to each meal. After surgery, the patient transitioned from enteral nutrition to oral intake, which was well tolerated. Serum sodium levels gradually improved.

F: female; GLIM: Global Leadership Initiative on Malnutrition; M: male; NRS-2002: Nutritional Risk Screening 2002; ONS: oral nutritional supplements.

Although only nine patients were identified as being at nutritional risk (NRS-2002 ≥3) in this study, simple group comparisons revealed that the risk group was significantly older, had lower BMI, and tended to experience longer hospital stays compared with the non-risk group (Table 3).

**Table 3.** Difference between the Risk Group and the Non-Risk Group.

	Risk group (n = 9)	Non-risk group (n = 539)	p-Value
Age (years)*	73.3 ± 16.4	34.5 ± 16.2	**<0.001**
Body mass index (kg/m^2^)*	19.4 ± 3.1	22.4 ± 4.2	**<0.001**
Length of hospital stay (days)*	15.4 ± 19.1	6.0 ± 4.1	**<0.001**
Sex (female), n (%)†	7 (77.8)	317 (58.8)	0.321

P-values for comparisons of study population characteristics between the risk group and the non-risk group were calculated using Student’s *t*-test. *Presented as the mean ± standard deviation. †Presented as n (%). The boldface indicates statistically significant values.

## Discussion

### Overview of study population and nutritional risk

This retrospective study assessed the implementation and clinical relevance of nutritional risk screening using the NRS-2002 in 548 patients admitted to the department of OMFS. The cohort was relatively young, with a mean age of 35 years, and most admissions involved moderately invasive procedures such as orthognathic surgery for jaw deformity and subsequent plate removal. Orthognathic surgery for jaw deformity in young patients constitutes the largest proportion of oral and maxillofacial procedures at our institution. Early oral intake after orthognathic surgery for jaw deformity has been reported to promote faster postoperative recovery ^(18)^. Previous studies showed an average body weight loss of about 4% within 2 weeks postoperatively, with total protein and serum albumin levels declining and recovering slowly. Longer operative time was associated with greater weight loss ^(19)^.

Surgical technique also influenced postoperative weight loss. Patients undergoing intermaxillary fixation (IMF) experienced significantly greater weight loss at 1 week compared with those treated with elastic traction. Early weight reduction associated with IMF may contribute to sustained postoperative weight loss ^(20)^. Furthermore, a report from Denmark demonstrated that patients undergoing orthognathic surgery for jaw deformity experienced an average weight loss of 2.79-3.56 kg. Greater weight loss was observed in men and in those who underwent bimaxillary procedures, and living arrangements as well as social support were suggested to influence weight loss at week 3 ^(21)^.

These findings indicate that preoperative nutritional screening is valuable even in young patients undergoing orthognathic surgery for jaw deformity, as it may support early recovery of nutritional status. Notably, the cohort mainly comprised otherwise healthy individuals with few chronic comorbidities, reflecting the typical profile of patients admitted for elective or semi-elective oral surgical procedures.

Overall, the prevalence of nutritional risk (NRS-2002 score ≥3) was very low at 1.6% (9/548), indicating that most patients were nutritionally stable. Among those identified as being at nutritional risk, key characteristics included advanced age, low BMI, and severe underlying diseases, such as MRONJ or mandibular osteomyelitis. These patients also tended to experience prolonged hospital stays, suggesting that nutritional risk is closely associated with both patient frailty and the complexity of surgical management. Notably, three of these nine patients met the GLIM criteria for malnutrition, highlighting the clinical relevance of early nutritional screening even in specialized surgical populations.

### Sex-based differences and disease distribution

Significant sex differences were observed in anthropometric measurements, including height, weight, and BMI, whereas age was comparable between men and women. Female patients had a significantly longer mean length of hospital stay compared with male patients (6.5 days vs. 5.6 days, p = 0.026) (Table 1). This disparity may be attributed to the higher proportion of orthognathic surgery for jaw deformity cases among women, as well as potential sex-based differences in postoperative recovery related to pain perception, inflammation, and nutritional intake.

The reasons for admission also differed by sex. Orthognathic surgery for jaw deformity, including plate removal, was more common in female patients, suggesting a tendency toward elective procedures motivated by aesthetic or functional considerations. Conversely, cystic lesions were significantly more frequent in male patients (31.7% vs. 13.6%, p < 0.001), consistent with previous reports documenting a male predominance in odontogenic keratocysts ^(22), (23), (24)^. Behavioral and biological factors may contribute to this pattern: men are generally less likely to seek timely dental care, which can lead to progression of dental caries and periodontal disease into chronic inflammatory lesions such as radicular cysts (Table 1). In addition, higher rates of smoking and alcohol consumption among men may impair tissue healing and promote chronic inflammation. Hormonal and immunological differences between sexes may also play a contributory role. For other conditions, such as tumors and inflammatory diseases, no significant sex differences were observed, indicating that sex-specific disparities are condition-dependent rather than universal across oral surgical patients.

### Age- and BMI-related disease trends

Age-specific trends revealed distinct patterns in the type of oral and maxillofacial conditions observed. Younger patients (10s-30s) predominantly underwent surgeries for jaw deformities, while middle-aged patients (40s-60s) more frequently required treatment for cysts, tooth extractions, and inflammatory diseases, reflecting the cumulative impact of chronic conditions, dental caries, and periodontal disease. In older adults (≥70 years), malignant tumors, mucosal diseases, and benign tumors were more common (Figure 1), highlighting the increasing influence of age-related neoplastic risk. Additionally, the “Other” category, including trauma and foreign body ingestion, was observed across all age groups, indicating that acute injuries are independent of age and often occur unpredictably.

BMI-specific analysis demonstrated that most patients fell within the normal range (18.5-25 kg/m^2^) (Figure 2), suggesting overall nutritional stability. Procedures for jaw deformities were concentrated among patients with BMI 18-24 kg/m^2^, corresponding to slender to normal body types. Although fewer patients had high BMI (≥30 kg/m^2^), inflammatory conditions, cysts, and tooth extractions still occurred, indicating that obesity may increase risk for chronic dental conditions and impair wound healing. Conversely, patients with low BMI (≤16 kg/m^2^) showed a higher prevalence of malignant tumors and mucosal diseases, suggesting a possible association with malnutrition, frailty, or cancer-related cachexia (Figure 2).

### Hospital stay and nutritional management

Length of hospitalization was closely associated with disease complexity. Short hospital stays (1-4 days) were generally related to minor procedures such as plate removal, tooth extractions, and cyst removal. Intermediate stays (5-10 days) were typically associated with mucosal diseases, benign tumors, and orthognathic surgery for jaw deformity, which required more intensive management. Long-term hospitalizations (>10 days) were most often observed in cases of malignant tumors and orthognathic surgery for jaw deformity, reflecting surgical complexity, the need for general anesthesia, and extensive postoperative care (Figure 3).

Among patients identified as at nutritional risk, seven were aged ≥73 years, often with low BMI. Three of the nine patients met the GLIM criteria for malnutrition. Nutritional interventions included ONS, administered in three patients (3 daily doses of 200 kcal each), and individualized dietary modifications, ranging from texture adjustments to flavor enhancement using umeboshi paste or seaweed paste. These approaches aimed to optimize oral intake while accommodating patient preferences and tolerances. Notably, one 85-year-old patient demonstrated a weight gain of 2.6 kg during hospitalization, highlighting the potential effectiveness of individualized nutritional support.

A distinctive feature of oral and maxillofacial surgical patients is that, despite oral dysfunction or pain-related eating difficulties, many maintain appetite and relatively good general condition. This is facilitated by comprehensive care, including specialized dental management of oral function, oral hygiene support by dental hygienists, and oversight by internists for systemic conditions. Such multidisciplinary coordination contributes to improved oral intake, postoperative recovery, and overall clinical outcomes.

### Clinical implications of nutritional screening

The findings demonstrate that, although overall nutritional risk is low in this cohort, NRS-2002 effectively identifies high-risk patients, particularly older individuals with low BMI and severe underlying diseases, including MRONJ and osteomyelitis. Group comparisons showed that these patients experienced longer hospital stays, reinforcing the importance of targeted nutritional interventions in vulnerable subgroups.

Nutritional challenges in OMFS are multifactorial, encompassing impaired mastication, dysphagia, pain, and local pathological conditions. In the present study, patients classified as at risk frequently presented with these severe conditions, necessitating tailored interventions such as ONS administration, progressive dietary modification, and flavor enhancement. Thus, although the prevalence of nutritional risk is low among younger patients, it becomes clinically significant in specific high-risk subgroups. These findings underscore the need for early and individualized nutritional assessment and intervention in perioperative care.

### Comparison with previous studies and screening recommendations

Our results align with prior studies in other surgical fields, supporting NRS-2002 as a practical first-line screening tool. The Japanese Society for Parenteral and Enteral Nutrition Therapy (JSPEN) recommends Malnutrition Universal Screening Tool (MUST) for the general adult population, NRS-2002 for hospitalized patients (with caution in those aged ≥75 years), and Mini Nutritional Assessment (MNA) for individuals aged ≥65 years ^(25)^. In our institution, the predominance of younger and underweight patients justified the use of NRS-2002 over MUST to avoid overestimation of nutritional risk.

Before August 2024, nutritional screening at our hospital followed a locally developed protocol. The adoption of NRS-2002, combined with GLIM criteria for malnutrition diagnosis, reflects global trends toward standardized screening and aligns with revisions in the Japanese medical fee schedule in 2024 ^(26)^. Reports on the use of NRS-2002 in oral surgery remain limited; thus, this study provides novel insights into its feasibility, accuracy, and clinical relevance in this specialized population.

Age- and disease-specific trends underscore the importance of targeted nutritional assessment. Jaw deformities were more common in younger women, while cysts and malignant tumors predominated in older men. This distribution highlights the necessity of age- and disease-tailored interventions, particularly given that malnourished hospitalized elderly patients have previously been reported to have a median survival of just 6 months ^(27)^, emphasizing the critical role of early nutritional support in improving outcomes.

Compared with general inpatients, the prevalence of nutritional risk and malnutrition in our cohort was markedly lower. Previous Japanese reports documented nutritional risk rates of 42% and malnutrition prevalence of 26% in university hospital inpatients ^(1)^, whereas our study found only 1.6% risk. This discrepancy likely reflects the younger, healthier population and highlights the need for caution when extrapolating these findings to older or more medically complex cohorts.

### Practical nutritional management and multidisciplinary care

In our institution, the first-line approach for patients with reduced oral intake is the use of ONS, such as fortified beverages or jelly-type products. When intake of staple foods (e.g., rice porridge) is insufficient, additional condiments such as seaweed paste or pickled plum paste are provided. If these measures fail to improve nutritional status or food consumption, individualized interventions are implemented, including assessment of food preferences, modification of food texture, and consideration of alternative nutritional support. During the observation period of this study (August 2024 to March 2025), ONS was administered to 14 patients, including those listed in Table 2, and supplementary condiments for staple foods were provided to 22 patients, also including those shown in Table 2.

Recent advances emphasize individualized interventions. The Effect of early nutritional support on Frailty, Functional Outcomes, and Recovery of malnourished medical inpatients Trial (EFFORT) demonstrated that, in medical inpatients at nutritional risk, systematic screening at hospital admission followed by the introduction of individualized nutritional support is effective in improving clinical outcomes ^(28)^. Similarly, in oral surgery, careful evaluation of multiple factors affecting intake―including psychological, social, and environmental determinants―is essential for effective intervention. When oral intake is insufficient, alternative routes such as enteral nutrition should be considered.

Preoperative hypoalbuminemia and prolonged surgical duration have been associated with postoperative complications ^(29)^. Historically, serum albumin was misused as a malnutrition marker in Japan; structured, quantitative assessments now represent the recommended standard ^(30), (31), (32)^. Perioperative nutritional management should not rely solely on preoperative serum albumin, but should incorporate comprehensive assessments such as the GLIM criteria.

### Limitations

Several limitations should be acknowledged. The study was retrospective, single-center, and spanned only 7 months. Nutritional risk was observed in only nine patients, precluding multivariate analysis and limiting statistical power. Interventions, including ONS and dietary modifications, were individualized rather than standardized, preventing systematic evaluation of efficacy. Comparisons with other screening tools (e.g., MUST, Subjective Global Assessment ［SGA]) were not performed, and the predominance of younger patients limits generalizability to frail or multimorbid populations. In addition, regional or seasonal factors may have influenced patient characteristics, introducing potential selection bias.

### Future directions

Future studies should adopt multicenter, prospective designs with longer observation periods (2-3 years), include larger and more diverse patient populations, and standardize nutritional interventions through NST protocols. Evaluating the relationship between NRS-2002 scores, clinical outcomes, and long-term nutritional status, including postdischarge weight changes and readmission rates, will provide robust evidence to guide perioperative nutritional management in OMFS.

### Conclusions

This study demonstrated that the overall prevalence of nutritional risk among OMFS inpatients was low; however, the NRS-2002 effectively identified older, underweight patients with severe conditions who were prone to prolonged hospital stays. These findings highlight the clinical relevance of early nutritional screening and the need for individualized interventions in high-risk subgroups. Incorporating standardized screening tools such as NRS-2002 into perioperative care may contribute to improved patient management and outcomes.

## Article Information

### Author Contributions

Hiroshi Kusunoki wrote the manuscript. Noriko Yasuda supervised the study from the perspective of a registered dietitian. Kaname Tsuji and Sho Mitsugi supervised the study from the perspective of an oral surgeon. Takayuki Kusunoki supervised the study from the perspective of a geriatric dentist. Haruka Shigemura supervised the study from the perspective of a nurse. Teruko Kawahara supervised the study from the perspective of a pharmacist. Miyu Matsui supervised the study from the perspective of a dental hygienist. Ayumi Saito supervised the study from the perspective of a clinical laboratory technician. Masanari Shimoda supervised the study from the perspective of an administrative staff member. Hideo Shimizu supervised the overall study.

Hiroshi Kusunoki and Noriko Yasuda contributed equally to this work.

### Conflicts of Interest

None

### Approval code Issued by the Institutional Review Board (IRB) and the Name of the Institution(S) That Granted the Approval

IRB approval number 111404 at Osaka Dental University.
